# Nutraceutical Interception of Cachexia: Grape-Derived Compounds as Pathophysiological Network Modulators

**DOI:** 10.3390/biology14091159

**Published:** 2025-09-01

**Authors:** Anderson Matheus Oliveira Haas Verdi, Mariana Lemos Rizzardi, Jaqueline Machado Soares, Dalton Luiz Schiessel, Dario Coletti, Marilia Cerqueira Leite Seelaender, Daniela Caetano Gonçalves

**Affiliations:** 1 Postgraduate Program in Nutrition, Federal University of São Paulo (UNIFESP), Baixada Santista Campus, Santos 11015-020, Brazil; anderson.verdi@unifesp.br (A.M.O.H.V.); marianarizzardi@gmail.com (M.L.R.); 2 Postgraduate Program in Community Development, Midwest State University (UNICENTRO), Irati Campus, Irati 11015-020, Brazil; nutrijaquesoares@gmail.com; 3 Department of Nutrition, Midwest State University (UNICENTRO), CEDETEG Campus, Guarapuava 85040-167, Brazil; dalton@unicentro.br; 4 Unit of Histology and Medical Embryology, Department of Anatomy, Histology, Forensic Medicine and Orthopedics, Sapienza University of Rome, 00161 Roma, Italy; dario.coletti@uniroma1.it; 5 Biological Adaptation and Ageing (B2A), Institut de Biologie Paris-Seine, CNRS UMR 8256, Inserm U1164, Sorbonne Université, 75005 Paris, France; 6 Cancer Metabolism Research Group, Department of Surgery and LIM 26-HCFMUSP, Faculty of Medicine, University of São Paulo, São Paulo 01246-903, Brazil; seelaender@usp.br; 7 Department of Biosciences, Federal University of São Paulo (UNIFESP), Baixada Santista Campus, Santos 11015-020, Brazil

**Keywords:** inflammation, muscle wasting, nutritional support, polyphenols, proteolysis

## Abstract

Cancer cachexia is a multifactorial syndrome characterized by metabolic dysregulation, inflammation, and progressive loss of skeletal muscle mass. Frequently observed in colorectal cancer patients, it is associated with poor clinical outcomes and reduced treatment tolerance. Current therapies provide limited benefit, underscoring the need for integrative approaches. Grape seed polyphenols, particularly oligomeric proanthocyanidins, have demonstrated potential to modulate catabolic signaling, mitochondrial dysfunction, and inflammatory responses involved in cachexia pathophysiology. This review integrates preclinical and clinical evidence on the use of grape seed-derived products, highlighting their effects on NF-κB and AMPK pathways, redox homeostasis, and gut–muscle axis. Furthermore, the nutritional composition and bioactive properties of grape seed flour—rich in fiber and phenolic compounds—are discussed in the context of nutraceutical applications. A clinical trial currently underway in Brazil aims to evaluate the effects of grape seed flour supplementation in colorectal cancer patients with cachexia. Altogether, grape-derived compounds offer a safe, low-cost, and mechanistically grounded strategy for improving nutritional status and resilience in oncological care.

## Simple Summary

Cancer-associated cachexia is a complex syndrome marked by involuntary weight loss, muscle wasting, and systemic inflammation, especially prevalent among patients with colorectal cancer. Despite advances in cancer treatment, there is still no standard intervention capable of reversing this debilitating condition. In this context, grape seed polyphenols have gained attention for their anti-inflammatory, antioxidant, and microbiota-modulating properties. This review explores the biological effects of these compounds and their potential to modulate molecular pathways involved in muscle degradation and inflammation. Supported by experimental and clinical evidence, grape seed flour and extract emerge as promising, sustainable, and safe interventions that may complement multimodal strategies to manage cachexia. The use of grape by-products represents not only a functional nutritional resource but also an environmentally responsible alternative in cancer supportive care.

## 1. Introduction

Colorectal cancer (CRC) remains one of the most prevalent malignancies worldwide, ranking third in incidence and second in cancer-related mortality, according to recent GLOBOCAN estimates [1]. It accounts for approximately 10% of all new cancer diagnoses annually [2], with notable geographic disparities that reflect variations in lifestyle, environmental exposure, and access to healthcare services [3]. In Brazil, projections from the National Cancer Institute (INCA) estimate nearly 45,000 new CRC cases per year between 2023 and 2025, underscoring its significant public health impact [4,5].

Despite advances in surgical techniques, chemotherapy, immunotherapy, and radiotherapy, standard CRC treatments often result in adverse effects such as mucositis, nausea, and anorexia [6]. When compounded by tumor-driven metabolic dysregulation, these factors contribute to a catabolic environment that predisposes patients to cancer-associated cachexia—a multifactorial syndrome affecting up to 80% of those with gastrointestinal malignancies [7].

Cachexia is defined by involuntary weight loss, systemic inflammation, anorexia and progressive skeletal muscle wasting [8]. Its pathogenesis involves more than reduced caloric intake; it encompasses profound metabolic alterations, frequently including insulin resistance, accelerated proteolysis, and increased resting energy expenditure [9]. These systemic changes compromise therapeutic efficacy, accelerate clinical decline, and are associated with decreased survival [10,11].

In this context, there is growing interest in integrative therapeutic strategies capable of modulating both inflammation and muscle metabolism in the scenario. Among bioactive dietary compounds with therapeutic potential, polyphenols derived from grape seeds (*Vitis vinifera* L.) have emerged as promising candidates due to pleiotropic effects on redox balance, cytokine regulation, and protein turnover mechanisms [12,13,14,15,16].

This review aims to explore the molecular mechanisms involved in colorectal cancer-associated cachexia and to discuss the therapeutic potential of polyphenols derived from grape seeds, with an emphasis on the *Vitis vinifera* variety, as a promising strategy in its treatment. Clinical implications and translational perspectives will also be discussed, highlighting the role of functional compounds in supportive oncology care.

## 2. Pathophysiology of Cancer-Associated Cachexia

Cancer-associated cachexia is a multifactorial and progressive syndrome characterized by systemic inflammation, metabolic dysregulation, and functional decline [17]. Unlike primary malnutrition, it results from complex interactions between tumor-derived factors, host immune and metabolic responses, and the adverse effects of oncological therapy [18]. These elements converge to disrupt homeostatic mechanisms and promote a persistent catabolic state.

The pro-inflammatory environment—driven by cytokines such as Tumor Necrosis Factor-alpha (TNF-α), Interleukin-6 (IL-6), and Interferon-gamma (IFN-γ)—triggers widespread tissue degradation, particularly in skeletal muscle and adipose tissue [19]. This inflammatory milieu accelerates proteolysis, impairs anabolic pathways, and induces energy imbalance, contributing to muscle wasting and lipolysis [20]. Oncological treatments, including chemotherapy and immunotherapy, exacerbate these effects by impairing mitochondrial function and increasing oxidative stress, further enhancing catabolic signaling [21,22].

Multiorgan involvement is a hallmark of cachexia. In skeletal muscle, mitochondrial dysfunction, reduced oxidative capacity, and anabolic resistance impair contractile function [23]. In the white (WAT) adipose tissue, increased lipolysis and impaired lipid clearance contribute to augmented circulating free fatty acids, hepatic steatosis, inflammation, and insulin resistance [24,25]. The liver, involved in the Cori cycle producing glucose de novo from tumor-derived lactate, prioritizes the synthesis of acute-phase proteins—such as C-reactive protein (CRP) and fibrinogen—at the expense of albumin and transferrin [26].

Gut microbiota alterations are also implicated in the pathophysiology of cachexia, with reductions in butyrate-producing bacteria compromising intestinal barrier integrity [27]. This facilitates microbial translocation and amplifies systemic inflammation through innate immune activation, particularly via Toll-like receptor 4 [28,29]. Additional consequences of cachexia include cardiac atrophy and hypothalamic dysregulation of appetite and energy homeostasis [30,31].

Given the complexity of the syndrome, diagnosis is often difficult and to render it an easier task Fearon et al. [8] proposed a three-stage model: (1) pre-cachexia (≤5% weight loss with early metabolic changes), (2) cachexia (>5% weight loss with systemic inflammation), and (3) refractory cachexia (>15% weight loss with multiorgan failure). This framework highlights the progressive nature of the syndrome and underscores the need for timely, multimodal interventions [8,31].

Cachexia is a common manifestation observed in various severe diseases, including cancer. As a result, a significant proportion of deaths associated with advanced-stage cancer can be attributed to cachexia rather than to the primary disease itself [32]. This underscores the critical importance of developing innovative and effective therapeutic approaches for the treatment of cachexia.

### 2.1. Molecular Signaling Pathways in Cancer-Associated Cachexia

Muscle alterations in cachexia involve multiple interconnected signaling pathways that regulate protein balance, mitochondrial function, and systemic inflammatory responses. The NF-κB pathway plays a central role in activating pro-catabolic genes, such as those related to the ubiquitin–proteasome system, and is stimulated by inflammatory cytokines, particularly TNF-α and interleukin-1 beta (IL-1β) [9,10,29]. The IL-6/STAT3 axis is also strongly associated with muscle atrophy, promoting the transcription of genes involved in protein degradation and contributing to anabolic resistance [9,33].

In contrast, the activated protein kinase (AMPK) pathway functions as an energy sensor, promoting metabolic adaptations that include the inhibition of anabolic processes under conditions of energy stress and the activation of mitochondrial biogenesis [10,34]. This effect is potentiated by the coactivation of Peroxisome proliferator-activated receptor gamma coactivator 1-alpha (PGC-1α), a master regulator of mitochondrial function and oxidative capacity in skeletal muscle [35,36,37,38]. In the context of cachexia, reduced PGC-1α expression compromises mitochondrial integrity, favoring metabolic dysfunction and the loss of lean mass.

An integrated understanding of these pathways is essential, as sustained activation of NF-κB and IL-6/STAT3, combined with suppression of AMPK and PGC-1α, creates a pro-catabolic, inflammatory, and energetically unfavorable environment for skeletal muscle, thereby perpetuating atrophy [9,10,33,34,35,36,37,38,39,40,41]. For clinical outcomes and potential therapeutic targets related to the modulation of these pathways, see Section 4.

## 3. Intracellular and Extracellular Signaling Pathways in Cancer Cachexia

Cancer-associated cachexia results from a coordinated disruption of signaling networks that maintain skeletal muscle and adipose tissue homeostasis [8,9,10]. Tumor-derived mediators and chronic systemic inflammation converge to activate catabolic cascades and suppress anabolic signaling, leading to tissue wasting and metabolic imbalance. The molecular mechanisms contributing to cancer-associated cachexia have been characterized and include pro-inflammatory and catabolic signaling, metabolic reprogramming, and impaired anabolic capacity. Key pathways, including NF-κB, IL-6/STAT3, AMPK, and PGC-1α, are discussed in detail in Section 2.1; here, we focus on additional mechanistic aspects such as the IL-6/STAT3–SOCS3 axis, the activation of the ubiquitin–proteasome system (UPS) and the autophagy–lysosome pathway, and the role of tumor-derived extracellular vesicles (EVs) in propagating catabolism.

Downstream, transcription factors including NF-κB, STAT3, and Smad2/3 upregulate atrogenes such as MuRF-1 and Atrogin-1, as well as autophagy-related genes like Beclin-1 [9,33,39]. IL-6/STAT3 signaling also contributes to anabolic resistance by impairing insulin/IGF-1 pathways through the induction of SOCS3 [9,33]. The UPS and autophagy–lysosome pathways are activated, with FoxO3 and AMPK playing pivotal roles by regulating atrogene expression and inhibiting mTORC1 [34,40,41,42].

Mitochondrial dysfunction remains central to this catabolic network, with impaired biogenesis, oxidative stress, and Dynamin-related protein 1 (DRP1)-mediated fission disrupting energy homeostasis and regenerative capacity [10,41]. Castro et al. [43] demonstrated that cancer-associated cachexia in patients induces profound morphological and functional alterations in skeletal muscle mitochondria, triggering autophagy and apoptosis pathways. Specifically, cachectic patients exhibited an increased intermyofibrillar mitochondrial area, elevated expression of Fis1, and higher levels of Microtubule-associated proteins 1A/1B light chain 3B, type II (LC3B II), Autophagy-related protein 5 (ATG5), and Autophagy-related protein 7 (ATG7), alongside activated caspases 8 and 9 and phosphorylated p53 [43].

This redundant signaling network contributes to the resistance of cachexia in the face of standard therapies. Therefore, interventions targeting IL-6R, ActRIIB, NF-κB, or Smad2/3 are under investigation [10,44]. In this context, grape seed polyphenols may be of interest as they have been proven to modulate NF-κB and AMPK pathways and oxidative stress [45].

The contemporary perspective on the role of reactive oxygen species (ROS) has evolved. Rather than being seen merely as harmful byproducts, ROS are now recognized as essential mediators of cellular signaling. Under physiological conditions, controlled fluctuations in oxidant levels constitute “oxidative eustress,” a beneficial state that triggers adaptive responses and maintains cellular function. This process operates within a homeodynamic range, a kind of “zone of equilibrium” [46,47].

## 4. Perspectives in the Treatment of Cancer-Related Cachexia

The search for an effective therapeutic approach to cachexia has been the focus of numerous studies. Although some of these have shown positive results in animal models, few have been successful in clinical trials. Recent studies have explored various pharmacological and multimodal interventions to address cachexia. Among candidate pharmacological strategies, several agents have been investigated for potential effects on inflammation, metabolism, and appetite regulation. A systematic review [48] assessed the efficacy of Non-Steroidal Anti-Inflammatory Drugs (NSAIDs), including Indomethacin, Ibuprofen, and Celecoxib, in treating cancer-associated cachexia. The review found insufficient evidence to recommend NSAIDs, with high risk of bias in most studies [48]. In a randomized, double-blind controlled trial with 70 patients, Mehrzad et al. [49] evaluated the use of Pentoxifylline (400 mg, 3 times/day) in cancer cachexia. No significant differences were found in weight or arm circumference between treatment and placebo groups at 4 or 8 weeks. While a temporary improvement in quality of life (QoL) was observed in week 4 in the Pentoxifylline group (QOL score: 2129.5 ± 536 vs. 1850.3 ± 459.9, *p* = 0.037), this benefit could not be detected after 8 weeks. Thus, the intervention showed no significant clinical benefit beyond a short-term improvement in QoL [49].

Interventional studies using appetite stimulants such as megestrol acetate (a synthetic derivative of progesterone) and eicosapentaenoic acid have shown improvements in patients’ appetite, but there is limited evidence of significant clinical improvement [50,51,52,53,54]. Systematic reviews by Ruiz Garcia et al. [31] and Pascual Lopez et al. [55] concluded that megestrol acetate may improve appetite and is associated with modest weight gain in cancer patients. Similarly, the systematic review by Talebi et al. [56] also reported short-term weight gain (≤8 weeks) and the use of megestrol acetate in combination with radio/chemotherapy as a concurrent treatment; however, the effect size was small.

A systematic review and meta-analysis by Rezaei et al. [57], including 1331 participants from five randomized controlled trials, evaluated the efficacy of anamorelin (ONO-7643, a selective ghrelin receptor agonist) in cancer cachexia. The pooled results showed significant increases in body weight (WMD: 1.56 kg, 95% CI: 1.20 to 1.92), lean body mass (WMD: 1.36 kg, 95% CI: 0.85 to 1.86), and fat mass (WMD: 1.02 kg, 95% CI: 0.51 to 1.53). Additionally, there was an increase in IGF-1 levels (WMD: 51.16 ng/mL, 95% CI: 41.42 to 60.90) and Insulin-like Growth Factor Binding Protein 3 (IGFBP-3) (WMD: 0.43 μg/mL, 95% CI: 0.17 to 0.68). Although overall appetite did not significantly improve in pooled analysis, subgroup analysis indicated a significant increase in appetite for patients receiving 100 mg/day of anamorelin (WMD: 0.59, 95% CI: 0.32 to 0.86) with no heterogeneity (I^2^ = 0%). Despite promising results, the analysis included only five short-term trials, with no assessment of functional or long-term outcomes. The effect on appetite was limited, and anamorelin has only been approved in Japan, with regulatory rejection in the U.S. and Europe due to marginal clinical benefits. Therefore, current evidence remains insufficient to support its widespread use [57].

Although early nutritional intervention (assessment and counseling, with the goal of achieving a high-protein diet, containing 1.5 g/kg/day) and multidisciplinary (Pharmacological treatment, physical exercise programs, and psychosocial interventions) care are essential to minimize the deterioration of nutritional status, there is currently no standardized and widely accepted intervention for the treatment of cancer-associated cachexia [53,54,58,59,60].

Among the most promising approaches, recent research has increasingly focused on multimodal interventions that combine nutritional, pharmacological, and physical strategies. To illustrate this approach, a phase 3 randomized trial investigated a multimodal intervention combining nutritional counseling with omega-3 supplementation, physical exercise, and NSAIDs in patients with cancer. The results showed that after 6 weeks, patients in the intervention group presented stabilized weight (mean change: 0.05 kg [SD 3.8]) compared to perceived weight loss in the standard care group (–0.99 kg [SD 3.2]; mean difference = –1.04 kg, 95% CI –2.02 to –0.06; *p* = 0.04). However, no significant differences were observed in muscle mass (–6.5 cm^2^ vs. –6.3 cm^2^; *p* = 0.93) or physical activity (step counts: –377.7 vs. –458; *p* = 0.89), indicating limited impact on functional endpoints [61].

Similarly, a phase 2 trial assessed a comprehensive multimodal care including ibuprofen, omega-3 fatty acids, Bojungikki-tang (a traditional immune-modulating agent), oral nutritional supplements, physical training, and psychiatric counseling. After 12 weeks, the intervention group showed a non-significant median increase in lean body mass (+91 g vs. –295 g; *p* = 0.3863) and no difference in handgrip strength (0.4 vs. 0.9; *p* = 0.2319), with no significant improvements in secondary outcomes either. The trial was terminated early due to low accrual and did not support clinical benefit of this intervention [62].

Additional evidence from Mantovani et al. [63], based on a multicenter randomized study with 332 patients with cancer-associated cachexia divided into five distinct intervention groups (arms), demonstrated that only the combined multimodal intervention (Arm 5—including medroxyprogesterone or megestrol acetate, eicosapentaenoic acid, L-carnitine, and thalidomide) resulted in significant improvements. Patients in this group showed a mean increase of 2.1 kg in lean body mass (*p* = 0.0148), a reduction in resting energy expenditure of approximately 160 kcal/day (*p* = 0.044), and a significant decrease in fatigue levels measured by Multidimensional Fatigue Symptom Inventory—Short Form (MFSI-SF) (*p* = 0.047). In contrast, the other intervention arms—Arm 1: hormonal agents only, Arm 2: eicosapentaenoic acid only, Arm 3: L-carnitine only, and Arm 4: thalidomide only—failed to produce statistically significant changes in lean body mass, resting energy expenditure, or fatigue [63].

Despite the diversity of interventions evaluated, clinical outcomes remain limited, especially in terms of reversing muscle wasting and systemic inflammation. As it is clear that mere calorie increase in the diet does not result in improved symptoms [8,9,64,65,66], the search for nutraceuticals with impact on inflammation and oxidative stress and little or no deleterious side-effects is mandatory. One promising alternative consists of grape seed extract, a low-cost, safe, and potentially effective natural product, with preclinical evidence of NF-κB and AMPK modulation and antioxidant effects [45].

## 5. Grape Seed Flour: Origin, Processing, and Nutritional Composition

The global wine and juice industry processes over 77 million tons of grapes annually, generating 20–30% of this mass as pomace. Within this byproduct, grape seeds represent 38–52% of dry weight and concentrate up to 70% of the pomace’s phenolic content [67,68,69]. This remarkable concentration transforms what was once considered waste into a valuable nutritional resource.

Grape seed flour (GSF) emerges as a paradigm of sustainable nutraceutical intervention, particularly for complex conditions like cachexia. The industrial processing of grapes creates a decentralized biorefinery system yielding this underutilized high-value coproduct [67,68,69]. The conversion of seeds into flour represents a clear case of agricultural upcycling—transforming surplus into a safe, cost-effective therapeutic agent that combines clinical viability with environmental responsibility.

Nutritional analysis reveals GSF contains 38–42% dietary fiber (predominantly insoluble), 10–12% high-quality protein, 14–16% lipids rich in tocopherols, and 5–8% polyphenols, with oligomeric procyanidins constituting 60–70% of the phenolic fraction [70,71,72,73,74]. Significant variation exists between cultivars; red varieties like Cabernet Sauvignon and Merlot contain 20–30% more polyphenols than white grapes, while juice production yields seeds with approximately 40% greater phenolic content than winemaking due to reduced fermentation leaching [75,76].

Post-processing methods critically impact quality preservation. Freeze-drying maintains 15% greater antioxidant activity compared to conventional methods, while nitrogen-modified atmosphere packaging reduces polyphenol degradation to just 0.5% monthly versus 2.5% under ambient conditions [77,78]. These processing advantages enhance the flour’s functional properties.

A crucial distinction exists between GSF and grape seed extract (GSE). While GSE, produced through solvent or supercritical CO_2_ extraction, contains over 90% polyphenols by dry weight [79,80], GSF retains the seed’s complete nutritional matrix through whole milling [70,81]. This fundamental difference dictates their respective applications: GSE serves targeted nutraceutical purposes, whereas GSF functions as a comprehensive food ingredient offering balanced nutritional support, albeit with lower polyphenol bioavailability.

Collectively, these properties establish GSF as a multifunctional intervention supporting muscle preservation, oxidative stress reduction and chronic disease prevention [70,71,72,73,74,75,76,77,78,82]. Its unique integration of nutritional density, clinical potential and production sustainability positions it as particularly valuable for cachexia management, bridging human health needs with environmentally responsible food systems.

## 6. Polyphenols and Fiber in Grape Seeds

Grape seeds are a rich source of both bioactive polyphenols and dietary fiber, offering complementary mechanisms that influence inflammation, oxidative stress, and gut–muscle axis regulation [81]. The polyphenolic fraction consists primarily of monomeric catechins, epicatechins, and a high concentration of oligomeric procyanidins, including B-type dimers, trimers, and higher-order polymers [81,83]. These compounds exhibit potent antioxidant activity, with an oxygen radical absorbance capacity (ORAC) up to five times greater than that of vitamin C [81,84,85]. The hydroxyl groups in flavanol structures facilitate hydrogen donation and stabilization of reactive oxygen species (ROS), thereby limiting oxidative damage in skeletal muscle cells [84,86]. Table 1 presents an adaptation of the nutritional composition of grape seed flour as evaluated by Martins et al. [87].

The dietary fiber in grape seeds, predominantly insoluble and lignin-bound, contributes to intestinal homeostasis and microbial modulation [88]. In an animal model supplemented with grape seed extract for 6 weeks, there was a 40% reduction in the total number of intestinal polyps, along with an 80% to 86% decrease in cell proliferation and a four-to eightfold increase in apoptosis [89]. Additionally, it increased colonic butyrate production—a short-chain fatty acid known to inhibit histone deacetylases (HDACs) and support gut barrier integrity [90]. These microbial shifts contribute to systemic anti-inflammatory effects and complement the molecular actions of grape polyphenols [85].

The combined presence of polyphenols and fiber gives grape seeds a multifunctional nutritional profile, capable of modulating dysbiosis, restoring epithelial integrity, and influencing host metabolism via gut-derived metabolites. These interactions may enhance the systemic efficacy of grape seed supplementation, particularly in chronic inflammatory and catabolic conditions such as cancer cachexia [85,88,89].

## 7. Digestion, Absorption, and Metabolism of Grape Seed Polyphenols

The scientific literature remains inconclusive regarding the digestion, absorption, metabolism, and excretion of polyphenols. These processes are highly complex, primarily due to the diverse structural conformations of polyphenol molecules and their varying degrees of polymerization and conjugation [91,92,93]. The polyphenols present in grape seeds—particularly oligomeric procyanidins—undergo complex digestive and metabolic processes that significantly influence their systemic bioactivity. Absorption efficiency varies by polymerization degree: dietary flavonoids generally have low bioavailability, and high-molecular-weight tannins are poorly absorbed in their original form due to their large molecular size [83,94].

In the oral cavity, some in vitro studies have included α-amylase in simulated digestion models. This enzyme, abundant in saliva, is effectively inhibited by proanthocyanidins, whose activity decreases after digestion. Low-molecular-weight polyphenols are partially absorbed in the gastrointestinal tract, whereas high-molecular-weight tannins and certain low-molecular-weight polyphenols are transported to the large intestine. There, they are either excreted or metabolized by enterobacteria. Consequently, the stomach plays only a minor role in polyphenol absorption. Phenolic compounds then pass to the stomach and cecum in rodents, where they are degraded into oligomers, monomers, and additional metabolites due to the acidic environment of the gastric system. However, it remains unclear whether gastric acidity affects the total polyphenol content [83,91,92,93,95].

Despite divergent findings across studies, it is hypothesized that the oral degradation of phenolic compounds varies according to their molecular type, interactions with other nutrients, and environmental factors related to food cultivation [83,92,94].

In general, most dietary polyphenols are not absorbed in their active forms. They must first undergo enzymatic transformation—such as methylation, glucuronidation, or sulfation—resulting in methylated and glycosylated metabolites that exhibit increased intestinal bioavailability. Alternatively, they may be fermented by the host’s gut microbiota to become absorbable. Small phenolics and their metabolites are actively transported via membrane carriers, whereas larger oligomers undergo passive diffusion or endocytosis [96]. Once modified, polyphenols are absorbed and transported to the liver via the portal vein, utilizing active, passive, or facilitated transport mechanisms [91,92].

Following hepatic metabolism, the resulting metabolites may be distributed to various organs, including the kidneys, lungs, and heart, where they may exert health-promoting effects. Nevertheless, only about 5–10% of total polyphenols are absorbed in the small intestine; the remainder accumulates in the large intestine and is excreted in the feces, resulting in low overall bioavailability. This limited absorption occurs because the body recognizes polyphenols as xenobiotics, thereby reducing their bioavailability compared to other nutrients [91,92].

## 8. Experimental Evidence on Grape Polyphenols and Their Applicability to Cancer-Related Cachexia

Preclinical studies involving grape-derived polyphenols—particularly grape seed extract (GSE) and grape seed flour (GSF)—have demonstrated pleiotropic effects on inflammatory (NF-κB), oxidative (Nrf2), and metabolic (AMPK/PGC-1α) pathways that are centrally involved in cachexia. However, the scarcity of models that integrate both tumor and muscle components—the so-called “tumor-gut-muscle axis”—requires a critical evaluation of the translational potential of these findings, especially in CRC, where intestinal dysbiosis and chemotherapy exacerbate systemic inflammation [97].

The study conducted by Wang et al. [98] used IL-10 knockout mice, which develop chronic intestinal inflammation, muscle atrophy, and progressive weight loss—features that mimic key aspects of CRC-associated cachexia. IL-10-mediated inflammation is particularly relevant in this cancer type, as chemotherapy (e.g., 5-FU) and dysbiosis increase intestinal permeability and bacterial translocation. The intervention with GSE (~0.2 mg/g/day) in drinking water, containing 95% flavanols, preserved 15% of muscle mass and reduced TNF-α levels in muscle tissue by 40%. The human-equivalent dose, estimated using standard interspecies conversion factors, is approximately 100 mg/day—a clinically feasible amount [99].

In contrast, Lambert et al. [100] used a model of muscle atrophy induced by lipopolysaccharide (LPS), where muscle loss is mediated by caspase-3—a catabolic pathway widely activated in CRC patients through parathyroid hormone-related protein (PTHrP). This protein is present in up to 60% of colorectal tumors, making the model mechanistically relevant. Daily administration of 50 mg/kg of a polyphenolic mixture (resveratrol, catechins, procyanidins, and anthocyanins) resulted in a 30% inhibition of caspase-3 activity and a 22% reduction in muscle loss. Although the model lacks a tumor component, the targeted pathway is directly implicated in cancer cachexia.

Complementarily, Myburgh et al. [101] evaluated the effects of procyanidins (20 mg/kg/day) in an acute muscle injury model. In the treated group, satellite cell activation occurred as early as 4 h after injury, compared to 7 days in the control group, along with a 50% reduction in local TNF-α. Although the dose is translational (~160 mg/day in humans), the acute nature of the model limits its relevance for a chronic and multifactorial condition like cachexia.

Similarly, Qin et al. [102] reported that resveratrol (450 mg/kg) increased PGC-1α expression and improved mitochondrial function by 35%. However, the human-equivalent dose (~3.6 g/day) is impractical. Moreover, resveratrol constitutes less than 0.1% of GSF content. In contrast, procyanidins—comprising 70–80% of GSF’s polyphenolic fraction—activate AMPK and indirectly PGC-1α, providing a more viable pathway for clinical application.

The study by Derry et al. [103] used a colorectal tumor model induced by azoxymethane (AOM) but focused exclusively on carcinogenesis. GSE supplementation (0.25–0.5%) led to a 55% reduction in tumor formation and a 65% decrease in PCNA expression. However, the lack of data on tumor–muscle cross-talk (e.g., IL-6, myostatin, and proteolysis) prevents inference about anti-cachectic effects. Future studies should integrate simultaneous assessments of tumor burden and muscle parameters to evaluate potential systemic benefits.

Although most studies use GSE, grape seed flour (GSF) offers relevant advantages, such as the presence of dietary fiber (38–42%), which favors gut microbiota modulation—an increasingly recognized mediator of the gut–muscle axis. This confers GSF the potential to act not only as an antioxidant and anti-inflammatory agent but also as a functional prebiotic. Table 2 presents the main experimental studies and their intervention methods.

## 9. Clinical Evidence and Safety Profile of Grape Seed Polyphenols

To date, clinical studies involving grape polyphenols have focused on populations with dyslipidemia, metabolic syndrome, and aging. While these conditions share inflammatory and oxidative features with cancer cachexia, they do not include muscle-related outcomes or cancer patients [80].

Kar et al. [80] conducted a clinical trial with 32 elderly participants (mean age 61.8 years) using 600 mg/day of GSE for 4 weeks. The intervention led to a 34% reduction in high-sensitivity C-reactive protein (hs-CRP) and a 28% increase in plasma glutathione. Both biomarkers are relevant for cachexia prognosis, as hs-CRP is considered a predictor of muscle loss and mortality in cancer. The absence of drug interactions with lipid-lowering agents suggests that GSE may be safe even in polypharmacy contexts, such as CRC treatment [104].

A meta-analysis of 15 randomized clinical trials with GSE reported reductions in fasting glucose, LDL cholesterol, triglycerides, and CRP (−0.8 mg/L), but no effects on muscle strength or body composition. Notably, baseline CRP levels in participants were lower than those typically observed in cachectic patients (≥10 mg/L), which limits the inference of direct clinical benefit. Future trials could explore higher doses or combination therapies (e.g., with omega-3 fatty acids) to enhance potential synergistic effects [80].

Unlike megestrol acetate—which acts as an orexigenic agent without preserving lean mass—or anamorelin—a ghrelin receptor agonist approved only in Japan—grape polyphenols exhibit a multimodal profile of action: they reduce inflammation via NF-κB inhibition, stimulate mitochondrial biogenesis via AMPK activation, and may restore the gut–muscle axis through dietary fiber intake.

Despite this potential, three critical gaps remain. First, no study has evaluated the use of GSE or GSF in the perioperative period—a phase of accelerated catabolism. Second, there is a lack of studies using validated muscle biomarkers, such as urinary 3-methylhistidine or CT-based muscle mass assessment. Third, vulnerable populations—such as older adults with CRC (≥70 years), who represent the majority of cachectic patients—remain underrepresented in clinical research. Table 3 summarizes human studies related to cachexia.

## 10. Potential Interventions with Grape Seed in the Management of Cachexia

The pathology of cachexia can be understood as a consequence of this balance being disrupted, wherein the system crosses a critical threshold of redox stress signaling (RST), plunging into a state of chronic “oxidative distress.” This distress is characterized by an imbalance favoring oxidants, which leads to the disruption of signaling and molecular damage. In this context, the function of grape polyphenols must be reinterpreted in a more sophisticated manner. Instead of acting as mere antioxidants, whose indiscriminate action could lead to “reductive distress”—an equally detrimental condition caused by an excess of reductants—it is more likely that they function as modulators of redox homeodynamics. Their action would be to attenuate the supraphysiological levels of oxidants that characterize distress, while simultaneously preserving the physiological fluctuations necessary for beneficial eustress signaling. This ability to restore redox balance, rather than simply suppressing all oxidative activity, may be the key mechanism behind their potential effects on preserving muscle mass and function [46,47].

Grape seed polyphenols, particularly oligomeric proanthocyanidins, have emerged as promising agents for modulating the multifactorial mechanisms underlying cancer-associated cachexia. A systematic review evaluated the effects of flavonoids on skeletal muscle mass, muscle function, and physical performance in adults with sarcopenia. Three studies demonstrated that flavonoids improved skeletal muscle mass, two showed enhanced muscle strength, and two reported better physical performance [106]. Another systematic review and meta-analysis assessed flavonoid intake in middle-aged and older adults with or without sarcopenia. The authors concluded that flavonoid supplementation led to significant gains in appendicular skeletal muscle mass and 6-min walk distance, though no effects were observed on muscle strength [107]. A separate meta-analysis found that polyphenol supplementation increased muscle mass but did not significantly impact muscle strength [108].

According to these reviews, phenolic compounds influence skeletal muscle anabolic pathways—though overlapping studies limit sample size. These compounds exhibit a broad range of bioactivities, targeting critical nodes in metabolic dysregulation, immune response, and mitochondrial integrity [81,109].

Mechanistically, proanthocyanidins inhibit nuclear translocation of NF-κB by stabilizing IκB-α and suppressing p65 phosphorylation, thereby reducing the expression of key pro-inflammatory cytokines such as TNF-α, IL-6, and IL-1β. Concurrently, they activate AMP-activated protein kinase (AMPK), a central metabolic sensor that promotes energy conservation and enhances autophagy regulation. This dual modulation restores energy balance and reduces muscle proteolysis [98,110,111].

At the epigenetic level, polyphenols can modulate microRNAs involved in inflammation and muscle atrophy—including the induction of tumor suppressors miR-34a, miR-424, and miR-503, as well as effects on miR-155 and miR-663 [112]. Furthermore, the high fiber content in grape seed flour can interact with the gut microbiota, stimulating butyrate production and inhibiting histone deacetylase (HDAC) activity. Although polyphenols themselves can also exert this effect, their action is more limited compared to that of the fibers [113,114].

Some studies suggest that phenolic compounds exert positive effects on mitochondrial function, contributing to both free radical control and the promotion of mitochondrial biogenesis [115]. This process involves a complex cascade of proteins, with the peroxisome proliferator-activated receptor gamma coactivator 1-alpha (PGC-1α) playing a central role. This coactivator, part of the peroxisome proliferator-activated receptor (PPAR) family, is highly expressed in tissues with high energy demands and integrates the activity of multiple transcription factors, stimulating energy metabolism and mitochondrial renewal [35,36,37,38].

In experimental models, PGC-1α overexpression mitigated mitochondrial dysfunction and induced mitochondrial proliferation, highlighting its therapeutic potential in energy-deficient conditions. Upregulation of this signaling pathway suggests that phenolic compounds may reverse mitochondrial dysfunction in skeletal muscle, supporting anabolic pathway maintenance in this tissue—despite some overlapping data across reviewed studies [35,37,38].

**Figure 1:**
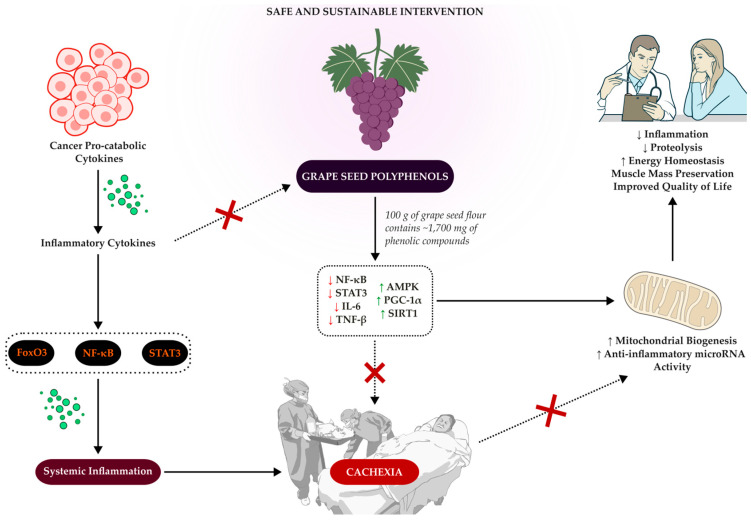
Mechanisms of Cancer Cachexia and Potential Modulatory Effects of Grape Seed Polyphenols.

Additionally, resveratrol stands out among phenolic compounds due to its ability to modulate multiple signaling pathways, including PGC-1α, SIRT1, mTOR, ERR-α, and AMPK [116]. Preclinical evidence highlights resveratrol’s efficacy in inducing SIRT1-mediated deacetylation of PGC-1α, which enhances the transcriptional activity of this coactivator in tissues such as neurons and liver in animal models. Furthermore, resveratrol activated AMPK, thereby increasing SIRT1 activation to boost PGC-1α-related transcription [35,37,38,117]. Figure 1 proposes possible mechanisms, addressed earlier, to halt the progression of cachexia.

## 11. Future Perspectives

Cancer cachexia remains a complex and treatment-refractory syndrome, characterized by profound metabolic alterations and progressive skeletal muscle loss. Despite advances in understanding its molecular basis, translation into effective treatments—particularly involving nutraceuticals—remains limited.

Further randomized controlled trials are needed to confirm the potential benefits and to establish evidence-based recommendations for clinical use. Importantly, the low bioavailability of polyphenols remains a major limitation for clinical translation. Approaches such as optimized formulation strategies and consideration of microbiota-mediated metabolism may enhance systemic exposure and therapeutic efficacy in cancer cachexia.

One major barrier is the methodological heterogeneity of clinical trials investigating bioactive compounds. Variations in extract standardization, dosage, intervention duration, and patient profiles hinder reproducibility and limit the formulation of evidence-based guidelines. Moreover, most studies are underpowered, with small and heterogeneous cohorts.

To address these limitations, our research group in Brazil is currently conducting a randomized, triple-blind, placebo-controlled clinical trial (registered at Brazilian Registry of Clinical Trials (ReBEC): RBR-5p6nv8b) to evaluate the efficacy of grape seed flour (GSF) in attenuating perioperative weight loss in patients with CRC at pre-cachexia or cachexia stages [118]. The trial includes participants aged 40–90 years, randomly allocated to receive either 8 g/day of GSF or a matching placebo for eight weeks (four weeks before and four weeks after surgery). The intervention is provided in standardized sachets and aims to preserve nutritional status during a period of high catabolic stress.

Inclusion criteria comprise adult patients with CRC scheduled for elective surgery, recent weight loss >5% in the previous six months or BMI < 20 kg/m^2^, and adequate clinical stability for the procedure. Exclusion criteria include inflammatory bowel disease, recent use of antioxidant supplements, severe renal or hepatic dysfunction, and any contraindication to fiber intake.

The trial aims to evaluate the attenuation of perioperative weight loss (primary outcome), as well as the preservation of fat-free mass, muscle strength, quality of life, anorexia, fatigue, and gastrointestinal symptoms (secondary outcomes). Standardized clinical monitoring includes inflammatory, metabolic, and nutritional biomarkers—all central to cachexia pathophysiology [14,119].

The chosen dose of 8 g/day of GSF provides approximately 136.25 mg of total phenolic compounds, including proanthocyanidins, which represent the dominant active fraction. This dosage is aligned with existing evidence demonstrating both safety and potential biological effects. For instance, Kar et al. [80] administered 600 mg/day of grape seed extract in elderly subjects for four weeks and reported significant anti-inflammatory and antioxidant effects. Park et al. [120] observed improvements in oxidative stress and blood pressure with 300 mg/day of grape seed proanthocyanidins in pre-hypertensive individuals, without adverse effects. Importantly, Sano et al. [121] conducted a dose-escalation trial in healthy adults using 1000 to 2500 mg/day of grape seed extract for four weeks and reported no clinically significant adverse events.

This ongoing trial represents a critical step in evaluating the translational potential of GSF in cancer cachexia, with a rigorous design, clearly defined outcomes, and alignment with existing safety data. Its results may inform future clinical recommendations and stimulate further research on functional foods and phenolic-rich interventions as part of multimodal strategies to mitigate cachexia-related deterioration in oncologic patients.

By integrating molecular, clinical, and nutritional data, this trial aims to provide robust translational evidence on the therapeutic potential of grape seed flour. The results may support the development of evidence-based nutritional strategies, contributing to a more integrative and personalized model of cancer care [61,122]. The main characteristics of our clinical trial, including study design, population, and outcomes, are summarized in Table 4.

## 12. Conclusions

Among emerging adjunctive strategies, grape seed flour stands out as a multifunctional compound with antioxidant, anti-inflammatory, and microbiota-modulating properties. While current clinical evidence is still preliminary, early findings suggest promising benefits in muscle preservation and overall patient resilience.

Overcoming key limitations—such as low bioavailability, study design heterogeneity, and lack of standardization—will require well-powered, multicenter trials integrating molecular, nutritional, and functional endpoints. Interdisciplinary collaboration among oncologists, clinical nutritionists, and translational researchers will be essential to integrate evidence-based nutraceuticals into routine cancer care.

In this context, grape seed-derived interventions offer a safe, scalable, and mechanistically sound strategy to mitigate cachexia progression and improve patient-centered outcomes.

## Figures and Tables

**Table 1:** Nutritional Information: Organic Grape Seed Flour.

	100 g
Energy value (kcal)	159
Total carbohydrates (g)	28
Total sugars (g)	3.4
Proteins (g)	8.6
Total fats (g)	1.3
Dietary fiber (g)	46
Phenolic compounds (mg)	1703
Hydrolysable tannins (mg)	307.7
Hydroxybenzoic acids and derivatives (mg)	153.8
Non-extractable proanthocyanidins (mg)	1287.1
Hydroxycinnamic acids (mg)	24.9
Anthocyanins (mg)	ND

**Table 2:** Preclinical studies with grape polyphenols in cancer cachexia models.

Study (Year)	Model Used	Intervention	Main Outcomes	Relevance to Cancer Cachexia
Wang et al. [98]	IL10-KO mice (chronic inflammation and muscle atrophy model)	GSE (0.2 mg/g/day in water)	15% preservation of muscle mass; ↓ TNF-α by 40%	Directly mimics colorectal cancer-associated cachexia
Lambert et al. [100]	LPS-induced muscle atrophy (caspase-3 mediated)	50 mg/kg of mixed polyphenols	↓ caspase-3 by 30%; ↓ muscle loss by 22%	Mechanistically relevant (PTHrP pathway activation in CRC
Myburgh et al. [101]	Acute muscle injury (gastrocnemius)	Grape seed procyanidins (20 mg/kg/day)	↑ satellite cell activation; ↓ local TNF-α by 50%	Limited due to acute, non-cachexia model
Qin et al. [102]	Post-injury muscle regeneration	Resveratrol (450 mg/kg)	↑ PGC-1α; ↑ mitochondrial function (35%)	Impractical dosage; not representative of cachexia
Derry et al. [103]	Colorectal cancer induced by AOM	GSE (0.25–0.5%)	↓ tumor formation (55%); ↓ PCNA (65%)	Focus on tumorigenesis, not cachexia parameters

**Table 3:** Clinical studies with grape polyphenols relevant to cachexia.

Study (Year)	Population	Intervention	Main Outcomes	Relevance to Cachexia
Kar et al. [80]	Elderly (mean age 61.8)	GSE (600 mg/day, 4 weeks)	↓ hs-CRP by 34%; ↑ glutathione by 28%	Biomarkers relevant to cachexia (inflammation, oxidative stress)
Foshati et al. [104]	Meta-analysis (15 trials)	GSE (various doses)	↓ CRP (by 0.8 mg/L); ↓ glucose, LDL, TG	No muscle outcomes; baseline CRP < cachexia levels
Malta & Gonçalves [105]	Colorectal cancer patients (perioperative)	GSF (8 g/day, 8 weeks)	Primary: ↓ weight loss; Secondary: muscle strength, inflammation, QoL	Ongoing RCT; first study focused on cancer cachexia

**Table 4:** Summary of the clinical trial design for grape seed flour supplementation in cancer cachexia.

**Item**	**Details**
Study Design	- Phase 2, randomized, placebo-controlled, triple-blind (participant, investigator, outcomes assessor) - Two-center trial (UNIFESP and partner site) - Superiority design (GSF vs. placebo)
Population	- CRC patients - Inclusion: Pre-cachexia/cachexia (Fearon et al. [8] criteria), age 40–90, BMI 18–30 kg/m^2^ - Exclusion: Metastatic disease, current antioxidant/anti-inflammatory use
Intervention	- GSF: 8 g/day (4 × 2 g capsules = 136 mg polyphenols + 3.7 g fiber) - Placebo: Matched corn starch capsules - Duration: 8 weeks (4 pre-op + 4 post-op)
Duration	- 8 weeks (4 preoperative weeks + 4 postoperative weeks), with 60-day follow-up for postoperative complications
Primary Endpoint	- Mean difference in body weight change (kg) from baseline to week 8 (± SD)
Secondary Endpoints	- Clinical: - Postoperative complications (Clavien–Dindo classification) - Adverse events (CTCAE v5.0) - Functional: - Handgrip strength (Jamar hydraulic dynamometer) - Quality of life (EORTC QLQ-C30) - Fatigue (FACIT-F scale) - Laboratory: - Systemic inflammation (CRP [mg/L], IL-6 [pg/mL]) - Muscle metabolism (urinary 3-methylhistidine) - Microbiological: - Gut microbiota (16S rRNA sequencing)
Safety Monitoring	- Daily adverse event tracking (nausea, diarrhea, allergies) - Hepatic/renal toxicity (AST, ALT, creatinine)
Ethics	- Approved by UNIFESP Ethics Committee (CAAE: 39368320.5.0000.5505) - Written informed consent obtained

## Abbreviations

The following abbreviations are used in this manuscript:

16S rRNA	16 Svedberg ribosomal RNA
ALT	Alanine Aminotransferase
AMPK	AMP-activated Protein Kinase
AST	Aspartate Aminotransferase
CAAE	Certificado de Apresentação para Apreciação Ética
CRC	Colorectal Cancer
CRP	C-reactive Protein
CTCAE	Common Terminology Criteria for Adverse Events
DRP1	Dynamin-related Protein 1
EORTC QLQ-C30	European Organization for Research and Treatment of Cancer—Quality of Life Questionnaire—Core 30
EPA	Eicosapentaenoic Acid
ERR-α	Estrogen-related Receptor Alpha
EVs	Extracellular Vesicles
FACIT-F	Functional Assessment of Chronic Illness Therapy—Fatigue Scale
GLOBOCAN	Global Cancer Observatory
GSE	Grape Seed Extract
GSF	Grape Seed Flour
HDAC	Histone Deacetylase
IFN-γ	Interferon gamma
IGF-1	Insulin-like Growth Factor 1
IGFBP-3	Insulin-like Growth Factor Binding Protein 3
IL-1β	Interleukin 1 beta
IL-6	Interleukin 6
INCA	Instituto Nacional de Câncer
MFSI-SF	Multidimensional Fatigue Symptom Inventory—Short Form
mTORC1	Mechanistic Target of Rapamycin Complex 1
NF-κB	Nuclear Factor kappa-light-chain-enhancer of activated B cells
NSAIDs	Non-Steroidal Anti-Inflammatory Drugs
ORAC	Oxygen Radical Absorbance Capacity
PCNA	Proliferating Cell Nuclear Antigen
PGC-1α	Peroxisome Proliferator-Activated Receptor Gamma Coactivator 1-alpha
PTHrP	Parathyroid Hormone-related Protein
QoL	Quality of Life
RNA	Ribonucleic Acid
ReBEC	Registro Brasileiro de Ensaios Clínicos
SIRT1	Sirtuin 1
Smad2/3	SMAD Family Members 2 and 3
SOCS3	Suppressor of Cytokine Signaling 3
STAT3	Signal Transducer and Activator of Transcription 3
TNF-α	Tumor Necrosis Factor alpha
UNIFESP	Universidade Federal de São Paulo
UPS	Ubiquitin-Proteasome System
WAT	White Adipose Tissue
WMD	Weighted Mean Difference
